# Neuroinflammation and psychiatric illness

**DOI:** 10.1186/1742-2094-10-43

**Published:** 2013-04-01

**Authors:** Souhel Najjar, Daniel M Pearlman, Kenneth Alper, Amanda Najjar, Orrin Devinsky

**Affiliations:** 1 Department of Neurology, New York University School of Medicine, 550 First Avenue, New York, NY, 10016, USA; 2 Geisel School of Medicine at Dartmouth, The Dartmouth Institute for Health Policy and Clinical Practice, 30 Lafayette Street, HB, Lebanon, NH, 7252, 03766, USA; 3 Department of Pathology, Division of Neuropathology, New York University School of Medicine, 550 First Avenue, New York, NY, 10016, USA; 4 Department of Psychiatry, New York University School of Medicine, New York, NY, USA; 5 New York University Comprehensive Epilepsy Center, 550 First Avenue, New York, NY, 10016, USA

**Keywords:** Neuroinflammation, Psychoneuroimmunology, Astrocyte, Microglia, Cytokines, Oxidative stress, Depression, Obsessive-compulsive disorder, Bipolar disorder, Schizophrenia

## Abstract

Multiple lines of evidence support the pathogenic role of neuroinflammation in psychiatric illness. While systemic autoimmune diseases are well-documented causes of neuropsychiatric disorders, synaptic autoimmune encephalitides with psychotic symptoms often go under-recognized. Parallel to the link between psychiatric symptoms and autoimmunity in autoimmune diseases, neuroimmunological abnormalities occur in classical psychiatric disorders (for example, major depressive, bipolar, schizophrenia, and obsessive-compulsive disorders). Investigations into the pathophysiology of these conditions traditionally stressed dysregulation of the glutamatergic and monoaminergic systems, but the mechanisms causing these neurotransmitter abnormalities remained elusive. We review the link between autoimmunity and neuropsychiatric disorders, and the human and experimental evidence supporting the pathogenic role of neuroinflammation in selected classical psychiatric disorders. Understanding how psychosocial, genetic, immunological and neurotransmitter systems interact can reveal pathogenic clues and help target new preventive and symptomatic therapies.

## Review

### Introduction

As biological abnormalities are increasingly identified among patients with psychiatric disorders, the distinction between neurological and psychiatric illness fades. In addition to systemic autoimmune diseases associated with psychiatric manifestations (for example, lupus) [1], more recently, patients with acute isolated psychosis were identified with synaptic autoimmune encephalitides (Table 1) [2-6]. These patients are often erroneously diagnosed with refractory primary psychotic disorders, delaying initiation of effective immune therapy (Table 1). Additionally, growing evidence supports the pathogenic role of anti-neuronal antibodies in neuropsychiatric disorders [7].

**Table 1:** Clinical features of anti-synaptic and anti-glutamic acid decarboxylase autoimmune encephalitides

**Clinical features**	**NMDAR, (NR1 subunit)**	**VGKC-complex (Kv1 subunit, LGI1, CASPR2)**	**AMPAR (GluR1, GluR2 subunits)**	**GABA**_**B**_**R (B1 subunit)**	**GAD (65 kDa)**
Age (years)	18 to 50	Less than 50	Less than 50	60 to 70	Less than 50
Sex (% female)	75%	66%	Greater than 50%	50%	Less than 50%
Etiology					
Paraneoplastic (%, and commonly occurring cancers)	9% to 56% have ovarian teratoma, predominately females less than 18 years of age	10% to 30%; low titers; SCLC; thymoma; CASPR2>>LGI-1	50% to 70%; SCLC, breast carcinoma; thymoma	50%; SCLC thymoma	Rarely associated with cancer
Nonparaneoplastic	Approximately 50%	70%; high titers	30% to 50%	50%	Frequent
Anatomical subtype					
Limbic encephalitis	Less common	Typical	Typical	Typical	Typical
Panencephalitis	Typical	Rare (involving basal ganglia)	Unclear	Unclear	Less common
CSF abnormal (%)	90%	40%	90%	80%	20%
Psychiatric Features	Common and pronounced: Anxiety, agitation, paranoid delusions, perceptual changes, erratic behavior, speech changes, severe psychosis	Agitation, anxiety, panic-attacks, depression, psychosis, hallucinations, delusions, delirium, confabulation	Atypical psychosis, which can be isolated	Paranoia, behavioral changes	Depression, atypical psychosis (case reports)
Neurological Features	Early features: seizures, cognitive/memory impairment; Late features: catatonia, orofacial and limb dyskinesia, dystonia, autonomic dysfunction, reduced level of consciousness, aphasia, central hypoventilation	LGI1: limbic encephalitis (more common): amnesia temporal lobe seizures, tonic seizures, and hypernatremia. Extrapyramidal symptoms (choreoathetosis) and extra-temporal (faciobrachial dystonic) seizures (less common). CASPR2: limbic encephalitis, Morvan's syndrome (neuromyotonia, REM disorder, insomnia, and autonomic dysfunction).	Memory impairment, temporal lobe seizures	Prominent temporal lobe seizures, memory impairment, concomitant glutamic acid decarboxylase autoantibodies	Stiff-person syndrome, cerebellar ataxia, cognitive/memory impairment, epilepsy (often mesial temporal)
Response to treatment	Highly responsive to immune therapy and removal of ovarian teratoma	Highly responsive to immune therapy	Moderately responsive to immune therapy	Moderately responsive to immune therapy	Often refractory to immune therapy
Relapse risk	20% often with psychiatric signs; may indicate tumor reoccurrence	Rarely relapses	Tendency to relapse (based on small case series)	Tendency to relapse (based on small case series)	Tendency to be chronic and relapse

*AMPAR*, 2-amino-3-(5-methyl-3-oxo-1,2-oxazol-4-yl)-propanoic acid receptor; *CASPR-2*, contactin associated protein 2; *CNS*, central nervous system; *CSF*, cerebrospinal fluid; *GABA*_*B*_*R*, gamma aminobutyric acid B receptor; *GAD*, glutamic acid decarboxylase; *LGI-1*, leucine-rich glioma inactivated-1; *NMDAR*, N-methyl-D-aspartate receptor; *REM*, rapid eye movements; *SCLC* small cell lung cancer; *VGKC*; voltage-gated potassium channel.

Separation of neurological and psychiatric disorders, supported by Descartes’s conception of the ‘mind’ as an ontologically distinct entity and by the reproducibility of neuropathological abnormalities, dominated medicine in the 19th and early 20th centuries [8]. Since then, an expanding collection of reproducible biological causes, from neurosyphilis, head trauma, stroke, tumor, demyelination and many others caused symptom complexes that overlapped with classic psychiatric disorders [9-11]. More recently, neuroinflammatory and immunological abnormalities have been documented in patients with classical psychiatric disorders.

Peripheral immune modulators can induce psychiatric symptoms in animal models and humans [12-19]. Healthy animals injected with pro-inflammatory IL-1β and tumor necrosis factor alpha (TNF-α) cytokines demonstrate ‘sickness behavior’ associated with social withdrawal [12]. In humans, injections of low-dose endotoxin deactivate the ventral striatum, a region critical for reward processing, producing anhedonia a debilitating depressive symptom [14]. Approximately 45% of non-depressed hepatitis C and cancer patients treated with IFN-α develop depressive symptoms associated with increased serum IL-6 levels [12,15,17,18].

Medical conditions associated with chronic inflammatory and immunological abnormalities, including obesity, diabetes, malignancies, rheumatoid arthritis, and multiple sclerosis, are risk factors for depression and bipolar disorder [10,12,13,15,17,18]. The positive correlation between these medical conditions and psy-chiatric illness suggests the presence of a widespread underlying inflammatory process affecting the brain among other organs [10,19,20]. A 30-year population-based study showed that having an autoimmune disease or a prior hospitalization for serious infection increased the risk of developing schizophrenia by 29% and 60%, respectively [16]. Further, herpes simplex virus, *Toxoplasma gondii*, cytomegalovirus, and influenza during pregnancy increase the risk of developing schizophrenia [16].

Peripheral cellular [21,22] (Table 2), and humoral immunological abnormalities [13,21-23] are more prevalent in psychiatric patients relative to healthy controls. In both pilot (n = 34 patients with major depressive disorder (MDD), n = 43 healthy controls) and replication studies (n = 36 MDD, n = 43 healthy controls), a serum assay comprising nine serum biomarkers distinguished MDD subjects from healthy controls with 91.7% sensitivity and 81.3% specificity; significantly elevated biomarkers for neuropsychiatric symptoms were the immunological molecules alpha 1 antitrypsin, myeloperoxidase, and soluble TNF-α receptor II [23].

**Table 2:** Summary of neuroinflammatory and immunological abnormalities observed in pure psychiatric disorders

**Abnormalities**	**Major depression**	**Bipolar disorder**	**Schizophrenia**	**OCD**
Genetics				
Concordance	37% to 38% [24]	40% to 70% [25]	40% to 50% [26]	80% to 87% [27]
GWAS genes	Tryptophan hydroxylase-1, BDNF, 5-HTTLPR, PBRM1 [24]	Tryptophan hydroxylase-2, Voltage-gated Ca^2+^ channel α1C, PBRM1, D22S278, ANK3 [25]	GABA_A_R B2 subunit, COMT, Neuregulin-1, DISC1 [26] HLA (B, C, DRA1, and DRB1; antigen presentation, autoimmunity) [28]	EAAT3 (SLC1A1) [27]
Immunologic genes	Proteasome β4 subunit (antigen processing) [21], T-box 21 (T cell differentiation) [21], IL-1 [29], TNF-α [29], G-765C (COX-2) [30], BDNF [24]	BDNF gene [31]; consistent with decreased serum BDNF levels	S100B [32,33]; consistent with increased brain and CSF S100B levels	TNF-α [34]
Astroglia				
Density	Decreased (highly reproducible) [35-41]; few exceptions [42,43]	Reduced or no change [37,44-46]	Reduced or no change [37-39,47-49][42,50,51]	Insufficient data
TDO, KYNA	…	KYNA is increased [52,53]	Both are increased [21,52]	…
Oligodendroglia				
Density	Decreased [54-58]	Decreased [54,56-65]	Decreased [54-58,66]	Insufficient data
Microglial activation				
Trait and State markers	Trait: no [53,67,68]; State (suicidal): yes [22,68]	Mixed data [53,68,69]	Trait: no [70]; State (suicidal): yes [22,68]	Insufficient data; yet, Hoxb8 (-/-) mice exhibit OCD-like behavior [71,72]
IDO, KMO	Both are increased [29]	…	Both are decreased [21],[73]	…
Quinolinic acid	Increased [53]	…	…	…
Lymphocytes				
T cells, T regs, B cells	T cells are decreased [15,74]; T regs are decreased [15,74]	T regs are increased [75]	T cells are decreased [76]; ‘CD4^+^**:** CD8^+^ ratio’ is decreased [76]; B cells are increased [21,76]	‘CD4^+^**:** CD8^+^ ratio’ is decreased [77] (normalized after SRI treatment)
EAAT				
EAATs 1,2 (astroglial)	Both are decreased in the DLPFC and ACC [78]	EAAT1 is increased, EAAT2 is decreased in PFC [79]	Both are increased in PFC [79-81], and thalamus [82]	…
EAATs 3,4 (neuronal)	EAAT4 is decreased in striatum [83]	Both are decreased in striatum [79]	EAAT3 is decreased in striatum [83]; Both are increased in PFC [79-81] and thalamus [82]	EAAT3 is decreased in CSTC circuitry [77,84]
Glutamate, GABA				
Post-mortem brain tissue	Glutamate and D-serine are increased in the frontal cortex [85,86]	Glutamate and D-serine are increased in DLPFC and hippocampus [86]. Increased glutamine in ACC/Parietal-OCC [78]	Glutamine synthetase is increased [87]	…
CSF, serum	Glutamate is increased in both; serum levels normalized after 5-week antidepressant course [85,86]; GABA is decreased in both [85,86]	…	…	CSF glutamate is increased [88]; normalized after one dose of ketamine (NMDAR antagonist)
^1^H MRS	Glutamate is increased [85,89] Decreased glutamine synthetase, glutamine, and GABA (ACC, PFC, DMPFC, VMPFC, amygdala, hippocampi; normalized with ECT and disease remission) [78]	GLX is increased in medial PFC (ACC, DLPFC, parietal-OCC, OCC, insula, hippocampus) [78,79]; independent of disease state.	Glutamate is decreased in medial PFC (including ACC); Increased glutamine synthetase, glutamine, and ‘glutamine**:**glutamate ratio’ in PFC [90]	GLX is increased in left caudate and OFC (normalized after successful SRI treatment); GLX is decreased in ACC [84]
Cytokines (serum)				
Phenotype	Proinflammatory are increased [91]	Proinflammatory are increased [92]; IL-1β, IL-1R, and IL-6 correlate with post-mortem brain mRNA expression [69]	Mixed data: antiinflammatory and/or proinflammatory, are increased [52,94,93]	Mixed data: TNF-α is increased or decreased; IL-6 is increased or no change; IL-1β is decreased [95]
Trait and State markers	Trait markers: TNF-α, IL-6, and sIL-2R are increased [91] State markers (suicidal): TNF-α and IL-6 are increased, and IL-2 is decreased [96].	Depressive state: IL-6 Euthymic state: IL-4 Manic state: IL-2, IL-4, IL-6 [92]	Trait makers: IFN-γ, TNF-α, IL-12, sIL-2R, IL-1RA, sIL-2R [93] State markers: IL-1β, IL-6, TGF-β [93]	Trait markers: mixed data LPS-induced: TNF-α and IL-6 are decreased [95]

*5-HTTLPR*, serotonin-transporter-linked polymorphic region; *ANK3*, ankyrin-3; *ACC*, anterior cingulate cortex; *BDNF*, brain-derived neurotrophic factor, *BPD*, bipolar disorder; *COMT*, catechol-O-methyl transferase; *COX-2*, cyclooxygenase 2; *CSF*, cerebrospinal fluid, *CSTC*, cortico-striatal-thalamic-cortico; *DLPFC*, dorsolateral prefrontal cortex; *DMPFC*, dorsomedial prefrontal cortex; *EAAT*, excitatory amino acid transporter: (EAATs 1 and 2 are expressed by astroglia, *EAAT3* is expressed intracellularly in the post-synaptic neurons, and *EAAT4* is expressed by Purkinje cells and frontal neurons); *GABA*, gamma aminobutyric acid; *GLX*^1^H MRS: (detectable glutamate, glutamine, gamma aminobutyric acid composite); *GWAS*, genome-wide association study (single nucleotide polymorphisms identified across the entire genome of those with a given disorder (fourth row); includes immunologic genetic abnormalities (fifth row) whenever applicable); ^1^*H MRS*, proton magnetic resonance spectroscopy; *HLA*, human leukocyte antigen; *IDO*, indoleamine-2,3-oxygenase; *IFN-*γ, interferon gamma; *IL*, interleukin, *IL-1RA*; interleukin 1 receptor antagonist; *KMO*, kynurenine monooxygenase; *KYNA*, kynurenic acid; *MDD*, major depressive disorder; *OCC*, occipital cortex; *OCD*, obsessive-compulsive disorder; *OFC*, orbitofrontal cortex; *PBRM1*, protein polybromo-1; *PFC*, prefrontal cortex; *SRI*, serotonin reuptake inhibitor; *T regs*, CD4^+^CD25^+^FOXP3^+^ T regulatory cells; *TDO*, tryptophan-2,3-dioxygenase, *TGF-*β, transforming growth factor beta; *TNF-*α, tumor necrosis factor alpha; *VMPFC*, ventromedial prefrontal cortex.

We first review the association between autoimmunity and neuropsychiatric disorders, including: 1) systemic lupus erythematosus (SLE) as a prototype of systemic autoimmune disease; 2) autoimmune encephalitides associated with serum anti-synaptic and glutamic acid decarboxylase (GAD) autoantibodies; and 3) pediatric neuropsychiatric autoimmune disorders associated with streptococcal infections (PANDAS) and pure obsessive-compulsive disorder (OCD) associated with anti-basal ganglia/thalamic autoantibodies. We then discuss the role of innate inflammation/autoimmunity in classical psychiatric disorders, including MDD, bipolar disorder (BPD), schizophrenia, and OCD.

### Neuropsychiatric disorders associated with autoimmunity

#### Systemic lupus erythematosus

Between 25% to 75% of SLE patients have central nervous system (CNS) involvement, with psychiatric symptoms typically occurring within the first two years of disease onset. Psychiatric symptoms may include anxiety, mood and psychotic disturbances [97]. Brain magnetic resonance imaging (MRI) is normal in approximately 42% of neuropsychiatric SLE cases [97]. Microangiopathy and blood–brain barrier (BBB) breakdown may permit entry of autoantibodies into the brain [97]. These antibodies include anti-ribosomal P (positive in 90% of psychotic SLE patients) [1], anti-endothelial cell, anti-ganglioside, anti-dsDNA, anti-2A/2B subunits of N-methyl-D-aspartate receptors (NMDAR) and anti-phospholipid antibodies [97]. Pro-inflammatory cytokines—principally IL-6 [97], S100B [97], intra-cellular adhesion molecule 1 [97] and matrix-metalloproteinase-9 [98] are also elevated in SLE. Psychiatric manifestations of SLE, Sjögren’s disease, Susac’s syndrome, CNS vasculitis, CNS Whipple’s disease, and Behçet’s disease were recently reviewed [1].

#### Neuropsychiatric autoimmune encephalitides associated with serum anti-synaptic and glutamic acid decarboxylase autoantibodies

Autoimmune encephalitides are characterized by an acute onset of temporal lobe seizures, psychiatric features, and cognitive deficits [2,3,99-108]. The pathophysiology is typically mediated by autoantibodies targeting synaptic or intracellular autoantigens in association with a paraneoplastic or nonparaneoplastic origin [3]. Anti-synaptic autoantibodies target NR1 subunits of the NMDAR [100,108,109], voltage-gated potassium channel (VGKC) complexes (Kv1 subunit, leucine-rich glioma inactivated (LGI1) and contactin associated protein 2 (CASPR2)) [101,102,106], GluR1 and GluR2 subunits of the amino-3-hydroxy-5-methyl-l-4-isoxazolepropionic acid receptor (AMPAR) [6,110,111] and B1 subunits of the γ-aminobutyric acid B receptors (GABA_B_R) [3,99,103]. Anti-intracellular autoantibodies target onconeuronal and GAD-65 autoantigens [2,3].

The inflammation associated with anti-synaptic autoantibodies, particularly NMDAR-autoantibodies, is typically much milder than that associated with GAD-autoantibodies or anti-neuronal autoantibodies related to systemic autoimmune disorders or paraneoplastic syndromes [2,107].

Although neurological symptoms eventually emerge, psychiatric manifestations, ranging from anxiety [2,3] to psychosis mimicking schizophrenia [2-6], can initially dominate or precede neurological features. Up to two-thirds of patients with anti-NMDAR autoimmune encephalitis, initially present to psychiatric services [5]. Anti-synaptic antibodies-mediated autoimmune encephalitides must be considered in the differential of acute psychosis [2-6]. Psychiatric presentations can include normal brain MRI and cerebrospinal fluid (CSF) analysis, without encephalopathy or seizures [2,3,5,6,107]. We reported a case of seropositive GAD autoantibodies associated with biopsy-proven neuroinflammation, despite normal brain MRI and CSF analyses, where the patient presented with isolated psychosis diagnosed as schizophrenia by Diagnostic and Statistical Manual of Mental Disorders, 4th Edition (DSM-IV) criteria [2]. Further, seronegative autoimmune encephalitides can also present with prominent neuropsychiatric disturbances, making diagnosis more elusive [107,112,113]. Psychiatric and neurological features associated with anti-synaptic and GAD autoantibodies are summarized in Table 1[1-6,99-108,114].

Serum anti-synaptic and GAD autoantibodies may occur in patients with pure psychiatric disorders [2,4,5,112,115-121]. In a prospective cohort of 29 subjects who met the DSM-IV criteria for schizophrenia, serum anti-NMDAR autoantibodies were found in three subjects, and anti-VGKC-complex autoantibodies were found in one subject [5]. Using more sensitive techniques to detect immunoglobulin G (IgG) NR1 autoantibodies in 100 patients with definite schizophrenia, no autoantibodies were identified [122]. However, this study did not assess autoantibodies targeting the NR2 subunit of NMDAR. Other studies reported significantly increased odds of elevated (≥90th percentile non-psychiatric control levels) NR2 antibody levels (odds ratio (OR) 2.78, 95% confidence interval (CI) 1.26 to 6.14, *P* = 0.012) among individuals with acute mania (n = 43), but not in chronic mania or schizophrenia [116].

#### PANDAS and pure obsessive-compulsive disorder associated with anti-basal ganglia/thalamic autoantibodies

OCD often complicates neurological disorders involving the basal ganglia including Sydenham’s chorea, Huntington’s disease and Parkinson’s disease. Anti-basal ganglia antibodies are implicated in Sydenham’s chorea [123]. PANDAS is characterized by acute exacerbations of OCD symptoms and/or motor/phonic tics following a prodromal group A β-hemolytic streptococcal infection. The pathophysiology is thought to involve cross-reactivity between anti-streptococcal antibodies and basal ganglia proteins [124]. The clinical overlap between the PANDAS and pure OCD suggests a common etiological mechanism [125].

Among a random cohort of 21 pure OCD patients, 91.3% had CSF anti-basal ganglia (*P* <0.05) and anti-thalamic autoantibodies (*P* <0.005) at 43 kDa [88], paralleling functional abnormalities in the cortico-striatal-thalamo-cortico circuitry of OCD subjects [84]. Another study documented that 42% (n = 21) of OCD pediatric and adolescent subjects had serum anti-basal ganglia autoantibodies at 40, 45, and 60 kDa compared to 2% to 10% of controls (*P* = 0.001) [7]. Anti–basal ganglia autoantibodies were detected in the sera of 64% of PANDAS subjects (n = 14), compared to only 9% (n = 2) of streptococcal-positive/OCD-negative controls (*P* <0.001) [126]. One study found no difference between the prevalence of anti-basal ganglia autoantibodies in OCD (5.4%, n = 4) versus MDD controls (0%) [127]; however, a limitation was the random use of rat cortex and bovine basal ganglia and cortex that might have limited the identification of seropositive cases.

The basal ganglia autoantigens are aldolase C (40 kDa), neuronal-specific/non-neuronal enolase (45 kDa doublet) and pyruvate kinase M1 (60 kDa)—neuronal glycolytic enzymes—involved in neurotransmission, neuronal metabolism and cell signaling [128]. These enzymes exhibit substantial structural homology to streptococcal proteins [129]. The latest study (96 OCD, 33 MDD, 17 schizophrenia subjects) tested patient serum against pyruvate kinase, aldolase C and enolase, specifically; a greater proportion of OCD subjects were sero-positive relative to controls (19.8% (n = 19) versus 4% [n = 2], *P* = 0.012) [130].

Yet, in the same study only one of 19 sero-positive OCD subjects also had positive anti-streptolysin O antibody titers, suggesting that in pure OCD the anti-streptolysin O antibody seronegativity does not exclude the presence of anti-basal ganglia autoantibodies.

In pure OCD, sero-positivity for anti-basal ganglia/thalamic antibodies is associated with increased levels of CSF glycine (*P* = 0.03) [88], suggesting that these antibodies contribute to hyperglutamatergia observed in OCD [84,88,131]. The improvement of infection-provoked OCD with immune therapies supports the pathogenicity of these autoantibodies [132]. A large NIH trial assessing the efficacy of intravenous immunoglobulin (IVIG) for children with acute onset OCD and anti-streptococcal antibodies is ongoing (ClinicalTrials.gov: NCT01281969). However, the finding of slightly higher CSF glutamate levels in OCD patients with negative CSF anti-basal ganglia/thalamic antibodies as compared to those with positive CSF antibodies suggests that non-immunological mechanisms may play role in OCD [84]. Other mechanisms, including cytokine-mediated inflammation (Table 2), are also hypothesized.

### Psychiatric disorders associated with innate inflammation

**Figure 1:**
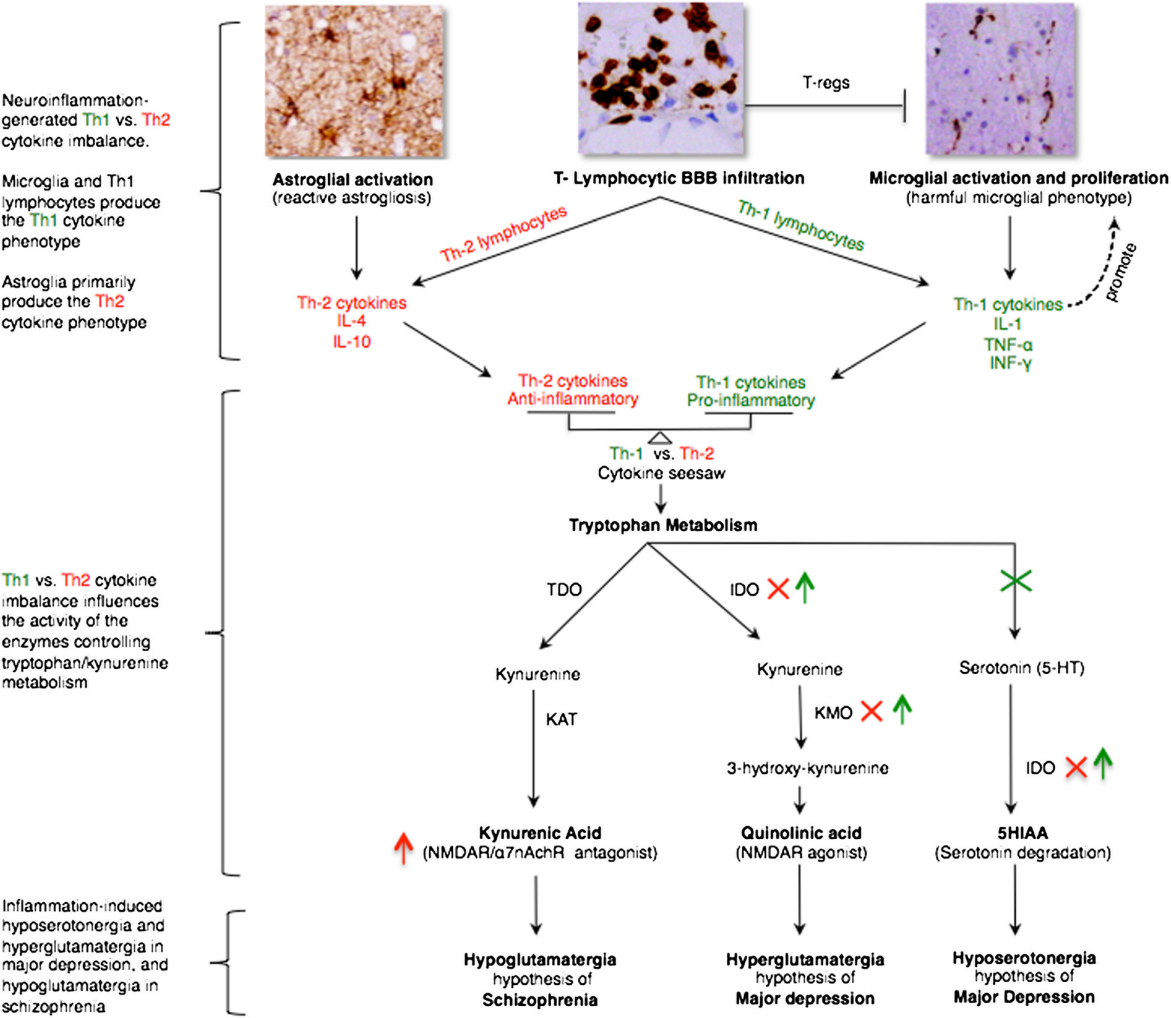
**Influence of the ‘Th1-Th2 cytokine seesaw’ generated by glial cells and T lymphocytes on tryptophan/kynurenine metabolism-mediated serotonergic and glutamatergic abnormalities in major depressive disorder and schizophrenia.** Influence of the ’Th1-Th2 seesaw’ generated by glial cells and T lymphocytes (first of three bracketed sections) on the enzymes controlling tryptophan/kynurenine metabolism (second of three bracketed sections) leading to serotonergic and glutamatergic abnormalities in major depressive disorder and schizophrenia (third of three bracketed sections). Microglial and astroglial IDO is the rate-limiting enzyme catalyzing the conversion of tryptophan to KYN and serotonin to 5HTT. KMO, which is solely expressed by microglia, is the rate-limiting enzyme catalyzing the conversion of KYN to 3-OH-KYN. TDO, which is solely expressed by astroglia, is the rate-limiting enzyme catalyzing the conversion of tryptophan to KYN. KAT, expressed primarily in astroglial processes, is the rate-limiting enzyme catalyzing the conversion of KYN to KYNA. The microglial enzymes IDO and KMO are upregulated by Th1 cytokines and downregulated by Th2 cytokines. An imbalance of the ‘Th1-Th2 seesaw’ shifts kynurenine catabolism either towards microglial quinolinic acid (NMDA agonist) as in major depressive disorder, or towards astroglial kynurenic acid (NMDA antagonist) as in schizophrenia. 5HIAA, 5-Hydroxyindoleacetic acid; α7nAchR, alpha 7 nicotinic acetylcholine receptors; BBB, blood–brain barrier; IDO, indoleamine-2,3-dioxygenase; IL, interleukin; IFN-γ, interferon gamma; KAT, kynurenine aminotransferase; KMO, kynurenine 3-monooxygenase; KYN, kynurenine; KYNA, kynurenic acid; NMDAR, N-methyl-D-aspartate receptor; TNF-α, tumor necrosis factor alpha; T regs, CD4^+^CD25^+^FOXP3^+^ T regulatory cells; TDO, tryptophan-2,3-dioxygenase; Th, T-helper.

**Figure 2:**
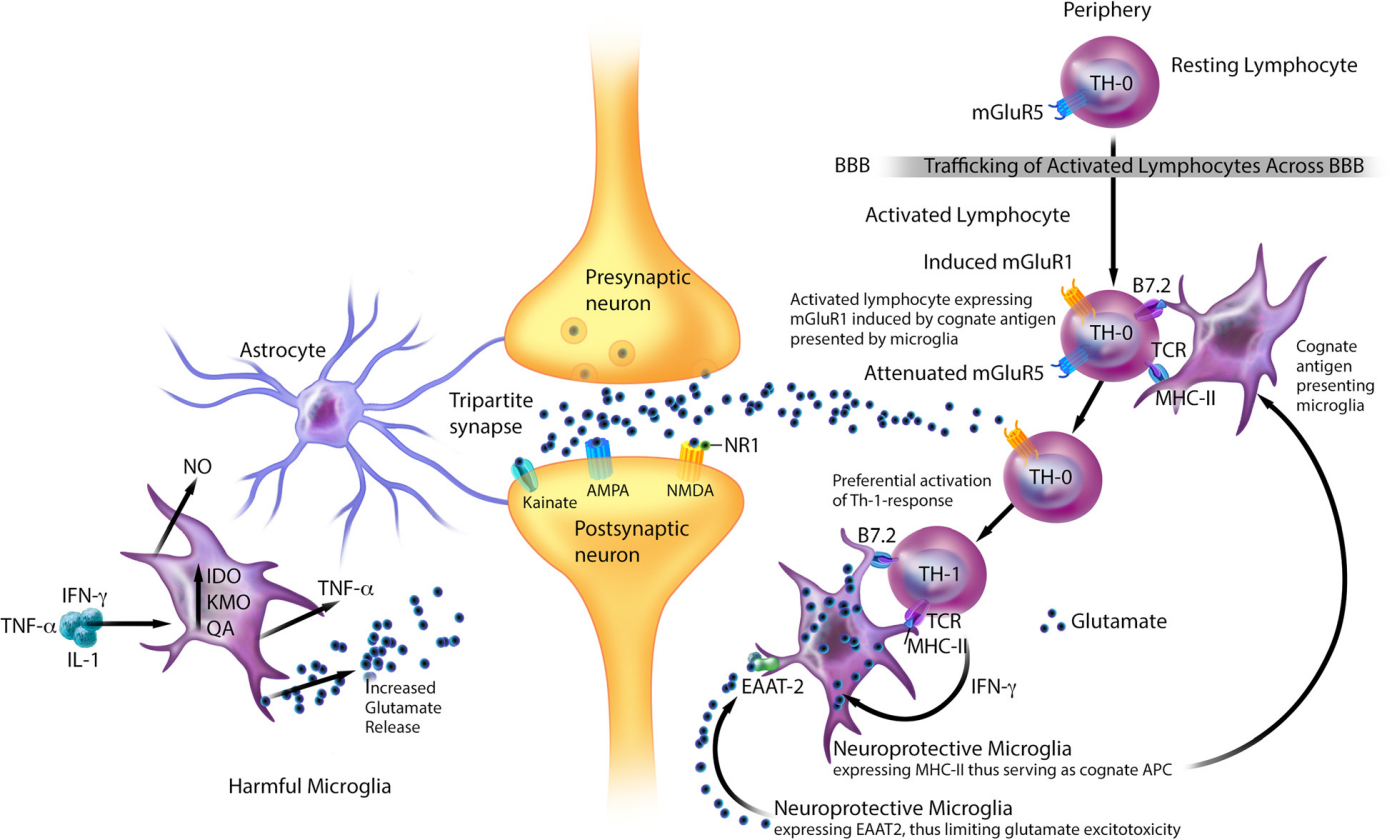
**Hypothesis of MDD: excess CNS glutamate may contribute to excess Th1- response promoting neuroprotective microglia.** Peripheral resting T lymphocytes constitutively express mGluR5. Activated T lymphocytes, but not resting T lymphocytes, can cross the BBB. In the animal models, the interaction between TCR of activated T lymphocytes and their cognate antigen presenting cells downregulates mGluR5 and induces mGluR1 expressions. Experimental data suggest that excess glutamate can bind to lymphocytic mGluR1 receptors, promoting production of Th1 cytokines. Hypothesis: In some MDD patients, parallel to experimental data, binding of excess CNS glutamate to induced lymphocytic mGluR1 receptors may contribute to an excess Th1 response, including IFN-γ. We further hypothesize that IFN-γ in a small quantity, similar to its in vitro effects on microglia, may induce microglial expression of MHC-II and EAAT-2, allowing microglia to serve as cognate antigen presenting cells and to provide glutamate reuptake function, thereby transforming harmful microglia into neuroprotective phenotype that participate in eliminating excess extracellular glutamate and reducing its excitotoxicity. Therefore, we hypothesize that excess Th1 response in some MDD patients is a double-edged sword; promoting harmful inflammation and serving as a beneficial counter-regulatory mechanism that may limit excess glutamate-related neuroexcitotoxicity? AMPA, 2-amino-3-(5-methyl-3-oxo-1,2-oxazol-4-yl)-propanoic acid; APC, antigen presenting cell; BBB, blood–brain barrier; CNS, central nervous system, EAAT, excitatory amino acid transporter; IDO, indoleamine-2,3-dioxygenase; IFN-γ, interferon gamma; IL, interleukin; KMO, kynurenine 3-monooxygenase; mGluR1/5, metabotropic glutamate receptors 1 and 5; MHC II, major histocompatibility complex class 2; NMDA, N-methyl-D-aspartate; NO, nitric oxide; NR1, glycine site; QA, quinolinic acid; TCR, T-cell receptor; Th, T-helper; TNF-α, tumor necrosis factor alpha.

Disorders of innate inflammation/autoimmunity occur in some patients with classical psychiatric disorders. We discuss innate inflammation-related CNS abnormalities—including glial pathology, elevated cytokines levels, cyclooxygenase activation, glutamate dysregulation, increased S100B levels, increased oxidative stress, and BBB dysfunction—in MDD, BPD, schizophrenia, and OCD. We also describe how innate inflammation may be mechanistically linked to the traditional monoaminergic and glutamatergic abnormalities reported in these disorders (Figures 1 and 2). The therapeutic role of antiinflammatory agents in psychiatric disorders is also reviewed.

#### Astroglial and oligodendroglial histopathology

Astroglia and oligodendroglia are essential to neural metabolic homeostasis, behavior and higher cognitive functions [54-56,133-136]. Normal quiescent astroglia provide energy and trophic support to neurons, regulate synaptic neurotransmission (Figure 2), synaptogenesis, cerebral blood flow, and maintain BBB integrity [134,136,137]. Mature oligodendroglia provide energy and trophic support to neurons and maintain BBB integrity, and regulate axonal repair and myelination of white matter tracts providing inter- and intra-hemispheric connectivity [54-56]. Both astroglia and oligodendroglia produce antiinflammatory cytokines that can down-regulate harmful inflammation [52,55].

In MDD, astroglial loss is a consistent post-mortem finding in functionally relevant areas, including the anterior cingulate cortex, prefrontal cortex, amygdala, and white matter [35-38,42-46,55,138-147], with few exceptions [42,43]. Post-mortem studies revealed reduced glial fibrillary acidic protein (GFAP)-positive astroglial density primarily in the prefrontal cortex [37,38] and amygdala [36]. A large proteomic analysis of frontal cortices from depressed patients showed significant reductions in three GFAP isoforms [39]. Although in one study that reported no significant glial loss, subgroup analysis revealed a significant decrease (75%) in GFAP-positive astroglial density among study subjects younger than 45 years of age [35]. A morphometric study similarly showed no changes in glial density in late-life MDD brains [148]. We hypothesize that the apparent absence of astroglial loss among older MDD patients may reflect secondary astrogliosis [35] that is associated with older age [42,50] rather than a true negative.

Animal studies are consistent with human studies showing astroglial loss in MDD. Wistar-Kyoto rats—known to exhibit depressive-like behaviors—revealed reduced astroglial density in the same areas as observed in humans [40]. Administration of the astroglial-toxic agent, L-alpha-aminoadipic acid, induces depressive-like symptoms in rats, suggesting that astroglial loss is pathogenic in MDD [41].

Post-mortem studies of MDD subjects documented reduced oligodendroglial density in the prefrontal cortex and amygdala [54-57,66], which may correlate with brain MRI focal white matter changes occasionally noted in some MDD patients [57]. However, microvascular abnormalities may also contribute to these changes [57].

In BPD, some studies demonstrate significant glial loss [138,143,149,150], while others do not [37,44-46]. These inconsistent findings may result from lack of control for: 1) treatment with mood stabilizers, because *post-hoc* analysis reported by some studies showed significant reduction in glial loss only after controlling for treatment with lithium and valproic acid [46]; 2) familial forms of BPD, as glial loss is particularly prominent among BPD patients with a strong family history [143]; and/or, 3) the predominant state of depression versus mania, as glial loss is frequent in MDD [35-38,42-46,55,138-147]. Whether astroglia or oligodendroglia account for the majority of glial loss is unclear; while proteomic analysis revealed a significant decrease in one astroglial GFAP isoform [39], several other post-mortem studies found either unchanged [36,37] or reduced GFAP-positive astroglial expression in the orbitrofrontal cortex [47], or reduced oligodendroglial density [54-56,58,59].

In schizophrenia, astroglial loss is an inconsistent finding [48,150]. While some studies showed no significant astroglial loss [42,50,51], several others found reduced astroglial density [37,38,43,44,48,49,151] and significant reductions in two GFAP isoforms [39]. Inconsistent findings may result from: 1) MDD comorbidity, which is often associated with glial loss; 2) age variation, as older patients have increased GFAP-positive astroglia [35,42,50]; 3) regional [150] and cortical layer variability [48]; 4) treatment with antipsychotic drugs, as experimental studies show both reduced [152] and increased [153] astroglial-density related to chronic antipsychotic treatment [70]; and 5) disease state (for example, suicidal versus non-suicidal behavior) [154]. Post-mortem studies documented oligodendroglial loss [54,56,60-65,148,155,156], particularly in the prefrontal cortex, anterior cingulate cortex, and hippocampus [148]. Ultrastructural examination of the prefrontal region showed abnormally myelinated fibers in both gray and white matter; both age and duration of illness were positively correlated with the white matter abnormalities [157].

In contrast to neurodegenerative disorders that are commonly associated with astroglial proliferation [136], psychiatric disorders are instead associated with either reduced or unchanged astroglial density [138]. The lack of increased glial density in early-onset psychiatric disorders [44,138] may reflect the slower rate of degenerative progression in psychiatric illnesses [138].

We postulate that degenerative changes associated with psychiatric disorders are subtler and not severe enough to provoke astroglial intracellular transcription factors that positively regulate astrogliosis, including signal transducer activator of transcription 3 and nuclear factor kappa B (NF-κB) [136].

While the majority of post-mortem studies focused on the alteration of glial density in MDD, BPD, and schizophrenia, others described alteration of glial cell morphology, with mixed findings. In MDD and BPD, glial size is either increased or unchanged [55]. One study found reduced glial size in BPD and schizophrenia but not in MDD [43]. A post-mortem study of depressed patients who committed suicide found increased astroglial size in the anterior cingulate white matter but not in the cortex [158]. One study in schizophrenic subjects found markedly decreased astroglial size in layer V of the dorsolateral prefrontal cortex, notwithstanding that astroglial density is double that of controls in the same layer [48]. The mixed results may partially reflect earlier studies of glial alterations in psychiatric illnesses that did not specify astroglia versus oligodendroglia [148].

Glial loss in psychiatric illnesses may contribute to neuroinflammation through several mechanisms, including abnormal cytokine levels (see *Cytokine* section), dysregulated glutamate metabolism (see *Glutamate* section), elevated S100B protein (see *S100B* section), and altered BBB function (see *Blood brain barrier* section), resulting in impaired cognition and behavior [44,45,54,133,159].

#### Microglial histopathology

Microglia are the resident immune cells of the CNS. They provide ongoing immune surveillance and regulate developmental synaptic pruning [160,161]. CNS injury transforms ramified resting microglia into activated elongated rod-shaped and macrophage-like phagocytic amoeboid cells that proliferate and migrate towards the site of injury along chemotactic gradients (that is, microglial activation and proliferation (MAP)) [161]. Human microglial cells express NMDARs that may mediate MAP leading to neuronal injury [162].

In MDD, BPD and schizophrenia, the results of post-mortem studies investigating the presence of MAP are mixed. Post-mortem studies revealed elevated MAP in only one out of five MDD subjects [67]. In some BPD disorder patients, increased human leukocyte antigen-DR-positive microglia displaying thickened processes were documented in the frontal cortex [69]. In schizophrenia, while some studies reported elevated MAP relative to controls, others showed no difference between groups [22,67,70]. In a post-mortem study assessing MAP in MDD and BPD; quinolinic acid-positive microglial cell density was increased in the subgenual anterior cingulate cortex and anterior midcingulate cortex of MDD and BPD patients who committed suicide relative to controls [53]. Post-hoc analysis revealed this increased MAP was solely attributable to MDD and not BPD, since the positive microglial immunostaining in MDD subjects was significantly greater than that in the BPD subgroup in both the subgenual anterior cingulate and midcingulate cortices, and since the microglia density was similar in both BPD and control groups [53]. A study comparing all three disorders (nine MDD, five BPD, fourteen schizophrenia, ten healthy controls) demonstrated no significant difference in microglial density across the four groups [68].

These mixed results may be attributed to variable microglial immunological markers used among different studies [70] and/or the failure to control for disease severity [22,53,68]. Notably, three post-mortem studies of MDD and schizophrenic subjects documented a strong positive correlation between MAP and suicidality in the anterior cingulate cortex and mediodorsal thalamus, independent of psychiatric diagnosis [22,53,68]. Thus, MAP may be a state rather than a trait marker for MDD and schizophrenia.

In OCD, animal models suggest that dysfunction and reduction of certain microglial phenotypes, such as those expressing the Hoxb8 gene, which encodes homeobox transcription factor, can cause OCD-like behavior [71,72]. Hoxb8 knockout mice exhibit excessive grooming behavior and anxiety in association with reduced microglial density [71,72]. This excessive grooming behavior resembles the behavioral characteristics of human OCD. Hoxb8 injection in adult Hoxb8 knockout mice reverses microglial loss and restores normal behavior [71,72]. The role of these specific microglial phenotypes in human OCD is unclear.

Experimental data suggest that MAP comprises distinctive harmful and neuroprotective phenotypes (Figure 2). Harmful microglia do not express major histocompatibility complex II (MHC-II) and, therefore, cannot act as antigen presenting cells (APC) [163,164]; they promote deleterious effects [17,69,165] through proinflammatory cytokine production, nitric oxide synthase signaling [17,166], promoting glial and BBB-pericyte/endothelial cyclooxygenase-2 (COX-2) expression [167], inducing astroglial S100B secretion (see *S100B* section), and microglial glutamate release [17,136,168,169]. Harmful microglia also secrete prostaglandin E-2 (PGE-2) that promotes proinflammatory cytokines production, which in turn increases PGE-2 levels in a feed-forward cycle [29]. Further, PGE-2 stimulates COX-2 expression, which mediates the conversion of arachidonic acid to PGE-2, setting up another feed-forward cycle [29].

Neuroprotective microglia by contrast can: 1) express MHC-II *in vivo* and *in vitro*[163,166] and act as cognate APC (Figure 2) [163,164,166]; 2) facilitate healing and limit neuronal injury by promoting secretion of antiinflammatory cytokines [17], brain-derived neurotrophic factor [17], and insulin-like growth factor-1 [166]; and 3) express excitatory amino acid transporter-2 (EAAT2) that eliminates excess extracellular glutamate [163,166], and promotes neuroprotective T lymphocytic autoimmunity (Figure 2) [163,164]. However, more studies are needed to confirm the contributory role of neuroprotective microglia to neuropsychiatric disorders in humans.

*In vitro* animal studies suggest that the ratio of harmful versus neuroprotective microglia can be influenced by the net effect of inflammatory counter-regulatory mechanisms [15,74,164,166]. These mechanisms include the number of neuroprotective CD4^+^CD25^+^FOXP3^+^ T regulatory cells ((T regs) Figure 1) [15,74,164,166] and brain cytokine levels; low IFN-γ levels may promote neuroprotective microglia (Figure 2) [166], whereas high levels can promote the harmful phenotype [166].

#### The role of cytokines

Proinflammatory cytokines include IL-1β, IL-2, IL-6, TNF-α and IFN-γ. They are secreted primarily by microglia, Th1 lymphocytes and M1 phenotype monocytes/macrophages (Figure 1) [15,170]. They promote harmful inflammation. Antiinflammatory cytokines include IL-4, IL-5 and IL-10. They are primarily secreted by astroglia, Th2 lymphocytes, T regs and M2 phenotype monocytes/macrophages [15,52,74]. They can limit harmful inflammation [15,74] by converting the proinflammatory M1-phenotype into the beneficial antiinflammatory M2-phenotype [15], and potentially by promoting the neuroprotective microglial phenotype [15,17,74,163,166]. The role of proinflammatory/antiinflammatory cytokines in psychiatric disorders is supported by several lines of evidence (Figure 1, Table 2) [15,17,29,52,74].

In MDD, the most recent meta-analysis (29 studies, 822 MDD, 726 healthy controls) of serum proinflammatory cytokines confirmed that soluble IL-2 receptor, IL-6 and TNF-α levels are increased in MDD (trait markers) [91], while, IL-1β, IL-2, IL-4, IL-8 and IL-10, are not statistically different from controls [91]. In a primary cytokine study comparing MDD subgroups (47 suicidal-MDD, 17 non-suicidal MDD, 16 health controls), both sera IL-6 and TNF-α were significantly higher, while IL-2 levels were significantly lower in MDD subjects who committed suicide relative to both other groups [96]. This finding suggests that IL-6 and TNF-α are also state markers of MDD [96]. The decrease of serum IL-2 levels associated with acute suicidal behavior may reflect increased binding to its upregulated receptor in the brain; parallel to the aforementioned meta-analysis showing increased soluble IL-2 receptor in MDD [91]. Studies investigating the clinical significance of cytokines in MDD showed that serum cytokine levels are elevated during acute depressive episodes [171,172] and normalized following successful, but not failed, treatment with antidepressants [17] and electroconvulsive therapy [29]; these findings suggest a possible pathogenic role for cytokines.

In BPD, serum cytokine alterations were summarized in a recent review; TNF-α, IL-6 and IL-8 are elevated during manic and depressive phases, whereas IL-2, IL-4 and IL-6 are elevated during mania [92]. Other studies showed that sera IL-1β and IL-1 receptor levels are not statistically different from healthy controls [92], although tissue studies documented increased levels of IL-1β and IL-1 receptor in the BPD frontal cortex [69].

In schizophrenia, results from studies investigating cytokine abnormalities are conflicting (Table 2). While some studies found both decreased serum proinflammatory (IL-2, IFN-γ) and increased serum and CSF antiinflammatory cytokines (IL-10) [52], others found elevated serum pro- and antiinflammatory cytokines, with a proinflammatory type dominance [22,173,174]. One cytokine meta-analysis (62 studies, 2,298 schizophrenia, 858 healthy controls) showed increased levels of IL-1R antagonist, sIL-2R and IL-6 [174]. However, this study did not account for the use of antipsychotics, which is thought to enhance proinflammatory cytokine production [52]. A more recent cytokine meta-analysis (40 studies, 2,572 schizophrenics, 4,401 controls) that accounted for antipsychotics, found that TNF-α, IFN-γ, IL-12 and sIL-2R are consistently elevated in chronic schizophrenia independent of disease activity (trait markers), while IL-1β, IL-6 and transforming growth factor beta positively correlate with disease activity (state markers)[173]. Cell cultures of peripheral blood mononuclear cells (PBMC) obtained from schizophrenic patients produced higher levels of IL-8 and IL-1β spontaneously as well as after stimulation by LPS, suggesting a role for activated monocytes/macrophages in schizophrenia pathology [175].

In OCD, results from a random survey of sera and CSF cytokines, and LPS-stimulated PBMC studies, are inconsistent [93-95,176-179]. There is a correlation between OCD and a functional polymorphism in the promoter region of the TNF-α gene [34], although low-powered studies did not confirm this association [180]. Therefore, the mixed results from studies documenting either increased or decreased TNF-α cytokine levels [93,176-178] may reflect their variable inclusion of the subset of OCD subjects with this particular polymorphism in their cohorts.

#### Cytokine response polarization in major depression and schizophrenia

Cytokine response phenotypes are classified as either proinflammatory Th1 (IL-2, IFN-γ) or antiinflammatory Th2 (IL-4, IL-5, IL-10) according to the immune functions they regulate. While Th1 cytokines regulate cell-mediated immunity directed against intra-cellular antigens, Th2 cytokines regulate humoral immunity directed against extra-cellular antigens [29,52]. Th1 cytokines are produced by Th1 lymphocytes and M1 monocytes whereas Th2 cytokines are produced by Th2 lymphocytes and M2 monocytes [29,52]. In the brain, microglia predominantly secrete Th1 cytokines, whereas astroglia predominately secrete Th2 cytokines [29,52]. The reciprocal ratio of Th1:Th2 cytokines, henceforth ‘Th1-Th2 seesaw’, is influenced by the proportion of activated microglia (excess Th1) to astroglia (excess Th2) and the interplay between activated T cells and excessive CNS glutamate levels that we hypothesized to favor Th1 response (Figure 2) [29,163,166].

The Th1-Th2 seesaw imbalance can influence tryptophan metabolism by altering its enzymes [21,52] thereby shifting tryptophan catabolism towards kynurenine (KYN) and KYN catabolism towards either of its two down-stream metabolites; microglia quinolinic acid that is Th1 response-mediated or astroglial kynurenic acid (KYNA) (Figure 1) that is Th2 response-mediated [21,29,170].

Tryptophan metabolism enzymes affected by Th1-Th2 seesaw include (Figure 1): indoleamine 2,3-dioxygenase (IDO) expressed by microglia and astroglia, the rate-limiting enzymes that mediate the conversion of tryptophan to KYN and serotonin to 5-hydroxyindoleacetic acid [21,29]. Kynurenine 3-monooxygenase (KMO), solely expressed by microglia, is the rate-limiting enzyme that converts KYN to 3-hydroxykynurenine (3-OH-KYN), which is further metabolized to quinolinic acid [21,29]. Tryptophan-2,3-dioxygenase (TDO), expressed solely by astroglia, is the rate-limiting enzyme that converts tryptophan to KYN [21,29]. Kynurenine aminotransferase (KAT), expressed primarily in astroglial processes, is the rate-limiting enzyme that mediates the conversion of KYN to KYNA [21,29].

Th1 cytokines activate microglial IDO and KMO, shifting microglial KYN catabolism towards quinolinic acid (NMDAR agonist) synthesis, while Th2 cytokines inactivate microglial IDO and KMO, shifting astroglial KYN catabolism towards TDO- and KAT-mediated KYNA (NMDAR antagonist) synthesis (Figure 1) [21,29].

Th1 and Th2 predominant immunophenotypes have been proposed for MDD and schizophrenia, respectively, based on peripheral, rather than CNS, cytokines patterns [52,173]. We believe that peripheral cytokines patterns are unreliable surrogate markers of those in the CNS. Indeed, peripheral cytokine levels can be influenced by many extra-CNS variables, which are not consistently controlled for in several of the peripheral cytokines studies, including: 1) age, body mass index, psychotropic medications, smoking, stress and circadian fluctuations; 2) the influence of disease activity/state on the production of selected cytokines synthesis [95,173]; and 3) the effects of psychotropic agents on cytokines production [52]. The short half-lives and the rapid turnover of serum cytokines [181] (for example, 18 minutes for TNF-α [182] versus 60 minutes for IL-10 [183]), may further limit the reliability of interpreting their levels measured from random sera sampling.

In MDD, there is a consensus that a proinflammatory Th1 immunophenotype response predominates (Table 2) [17,29]. High levels of quinolinic acid in post-mortem MDD brains [53], suggest the presence of an upregulated Th1 response (Figure 1) [21,29]. Elevated CNS quinolinic acid can promote calcium influx mediated apoptosis of human astroglia [184], which hypothetically may blunt the astroglia-derived Th2 response [29], tipping Th1 versus Th2 seesaw balance in favor of the microglial Th1 response. CNS hyposerotonergia [29] adds further support to an excess Th1 response, which is shown to reduce CNS serotonin synthesis [185] and to increase its degradation (Figure 1) [21,29].

CNS hyperglutamatergia may also contribute to an excess Th1 response in the brain (Figure 2). An *in vitro* study suggests that the peripheral resting T lymphocytes constitutively express metabotropic glutamate receptor 5 (mGluR5) [164], whose binding to glutamate inhibits lymphocytic IL-6 release, thereby downregulating auto-reactive T-effector cell proliferation [164]. Activated T lymphocytes, but not resting T lymphocytes, can cross the BBB [37].

Experimental data suggest that the interaction between T cell receptors of activated T lymphocytes and their cognate antigen presenting cells can downregulate mGluR5 and induce mGluR1 expressions [164]. In animal models, binding of excess glutamate to lymphocytic mGluR1 receptors promotes production of Th1 cytokines, including IFN-γ [164].

We hypothesize that in some MDD patients, parallel to experimental data [164], the binding of excess CNS glutamate to induced lymphocytic mGluR1 receptors may contribute to an excess Th1 response, including IFN-γ (Figure 2). We speculate that IFN-γ in a small quantity, similar to its *in vitro* effects on microglia [166], may induce microglial expression of MHC-II and EAAT2 [163,166], allowing microglia to serve as cognate antigen presenting cells and to provide glutamate reuptake function [163,164,166], thereby transforming harmful microglia into neuroprotective phenotype [163,166] that participate in eliminating excess extracellular glutamate [163,164,166]. Therefore, we also hypothesize that excess Th1 response in subgroups of MDD patients is a double-edged sword, promoting harmful inflammation and serving as a beneficial counter-regulatory mechanism that may limit excess glutamate-related neuroexcitotoxicity (Figure 2).

In schizophrenia, while some peripheral cytokine studies suggest the predominance of an antiinflammatory Th2 immunophenotype/response [52], others refute this [173,174]. However, we agree with the authors who hypothesized that the Th2 response is the dominant phenotype in schizophrenia [52]. Elevated brain, CSF, and serum levels of KYNA [21,52] suggest downregulation of microglial IDO and KMO, which is a function of Th2 response that shifts astroglial KYN catabolism towards KYNA synthesis (Figure 1) [21,52]. Reduced KMO activity and KMO mRNA expression in post-mortem schizophrenic brains [73] is consistent with excess Th2 response (Figure 1). Increased prevalence of Th2-mediated humoral immunity abnormalities in subgroups of schizophrenia patients—as evidenced by increased B cell counts [21,76], increased production of autoantibodies including antiviral antibodies [76] and increased immunoglobulin E [52]—adds further support to the Th2 response dominance hypothesis.

#### Neuroinflammation and CNS glutamate dysregulation

Glutamate mediates cognition and behavior [186]. Synaptic glutamate levels are regulated by high-affinity sodium-dependent glial and neuronal EAATs, namely, the X_AG_- system responsible for glutamate reuptake/aspartate release [137,164] and the sodium-independent astroglial glutamate/cystine antiporter system (X_c_-) responsible for glutamate release/cystine reuptake [164]. Astroglial EAAT1 and EAAT2 provide more than 90% of glutamate re-uptake [79].

Neuroinflammation can alter glutamate metabolism and the function of its transporters [15,29,187,188], producing cognitive, behavioral, and psychiatric impairments [15,21,29,79,186,188,189]. Abnormalities of EAATs function/expression and glutamate metabolism in MDD, BPD, schizophrenia, and OCD are summarized in Table 2.

In MDD, there is evidence for cortical hyperglutamatergia (Table 2). Cortical glutamate levels correlated positively with the severity of depressive symptoms, and a five-week course of antidepressants decreased serum glutamate concentrations [85,86]. A single dose of ketamine, a potent NMDAR antagonist, can reverse refractory MDD for a week [17,21,29,85]. Excess CNS glutamate levels can induce neurotoxicity-mediated inflammation [163,164,188], including a proinflammatory Th1 response (Figure 2) [164].

Limited *in vitro* evidence suggests that inflammation/proinflammatory cytokines can increase CNS glutamate levels [188] in a feed-forward cycle through several potential mechanisms: 1) proinflammatory cytokines can inhibit [15,17,168] and reverse [45,137] astroglial EAAT-mediated glutamate reuptake function; 2) proinflammatory cytokines can enhance microglial quinolinic acid synthesis [53], which has been experimentally shown to promote synaptosomal glutamate release [15,17,29,190]; 3) increased COX-2/PGE-2 and TNF-α levels can induce calcium influx [137], which, based on *in vitro* data, may increase astroglial glutamate and D-serine release [191]; and 4) activated microglia can express excess X_c_- antiporter systems that mediate glutamate release [164,192].

In schizophrenia, prefrontal cortical hypoglutamatergia [87,90,193,194] (Table 2) and reduced NMDAR functionality are found [5]. Recent H^1^ magnetic resonance spectroscopy (MRS) meta-analysis (28 studies, 647 schizophrenia, 608 control) confirmed decreased glutamate and increased glutamine levels in the medial frontal cortex [90]. The contributory role of inflammation to hypoglutamatergia is not proven. Elevated KYNA synthesis in schizophrenia brains [21,52], typically a function of Th2 response (Figure 1), can inhibit NR1 subunit of NMDAR and alpha 7 nicotinic acetylcholine receptor (α7nAchR) [195], leading to decreased NMDAR function and reduced α7nAchR-mediated glutamate release [195].

In BPD and OCD, data suggest CNS cortical hyper-glutamatergia in both disorders (Table 2) [78,84,88,131]. The contribution of inflammation (BPD and OCD) and autoantibodies (OCD)[7,77,84,88,130] to increased CNS glutamate levels requires further investigation.

#### The role of S100B

S100B is a 10 kDa calcium-binding protein produced by astroglia, oligodendroglia, and choroid plexus ependymal cells [196]. It mediates its effects on the surrounding neurons and glia via the receptor for advanced glycation end-product [196]. Nanomolar extracellular S100B levels provide beneficial neurotrophic effects, limit stress-related neuronal injury, inhibit microglial TNF-α release, and increase astroglial glutamate reuptake [196]. Micromolar S100B concentrations, predominantly produced by activated astroglia and lymphocytes [196,197], have harmful effects transduced by receptor for advanced glycation end product that include neuronal apoptosis, production of COX-2/PGE-2, IL-1β and inducible nitric oxide species, and upregulation of monocytic/microglial TNF-α secretion [21,196,198].

Serum and, particularly, CSF and brain tissue S100B levels are indicators of glial (predominantly astroglial) activation [199]. In MDD and psychosis, serum S100B levels positively correlate with the severity of suicidality, independent of psychiatric diagnosis [200]. Post-mortem analysis of S100B showed decreased levels in the dorsolateral prefrontal cortex of MDD and BPD, and increased levels in the parietal cortex of BPD [196].

Meta-analysis (193 mood disorder, 132 healthy controls) confirmed elevated serum and CSF S100B levels in mood disorders, particularly during acute depressive episodes and mania [201].

In schizophrenia, brain, CSF and serum S100B levels are elevated [199,202]. Meta-analysis (12 studies, 380 schizophrenia, 358 healthy controls) confirmed elevated serum S100B levels in schizophrenia [203]. In post-mortem brains of schizophrenia subjects, S100B-immunoreactive astroglia are found in areas implicated in schizophrenia, including anterior cingulate cortex, dorsolateral prefrontal cortex, orbitofrontal cortex and hippocampi [154]. Elevated S100B levels correlate with paranoid [154] and negativistic psychosis [204], impaired cognition, poor therapeutic response and duration of illness [202]. Genetic polymorphisms in S100B [32] and receptor for advanced glycation end-product genes in schizophrenia cohorts (Table 2) [32,33,205] suggest these abnormalities are likely primary/pathogenic rather than secondary/biomarkers. Indeed, the decrease in serum S100B levels following treatment with antidepressants [201] and antipsychotics [196] suggests some clinical relevance of S100B to the pathophysiology of psychiatric disorders.

#### Neuroinflammation and increased oxidative stress

Oxidative stress is a condition in which an excess of oxidants damages or modifies biological macromolecules such as lipids, proteins and DNA [206-209]. This excess results from increased oxidant production, decreased oxidant elimination, defective antioxidant defenses, or some combination thereof [206-209]. The brain is particularly vulnerable to oxidative stress due to: 1) elevated amounts of peroxidizable polyunsaturated fatty acids; 2) relatively high content of trace minerals that induce lipid peroxidation and oxygen radicals (for example, iron, copper); 3) high oxygen utilization; and 3) limited anti-oxidation mechanisms [206,207].

Excess oxidative stress can occur in MDD [206], BPD [206,207], schizophrenia [207,209], and OCD [206,208]. Peripheral markers of oxidative disturbances include increased lipid peroxidation products (for example, malondialdehyde and 4-hydroxy-2-nonenal), increased nitric oxide (NO) metabolites, decreased antioxidants (for example, glutathione) and altered antioxidant enzyme levels [206,207].

In MDD, increased superoxide radical anion production correlates with increased oxidation-mediated neutrophil apoptosis [206]. Serum levels of antioxidant enzymes (for example, superoxide dismutase-1) are elevated during acute depressive episodes and normalize after selective serotonin reuptake inhibitors (SSRIs) treatment [206]. This suggests that in MDD, serum antioxidant enzyme levels are a state marker, which may reflect a compensatory mechanism that counteracts acute increases in oxidative stress. [206]. In schizophrenia by contrast, CSF soluble superoxide dismutase-1 levels are substantially decreased in early-onset schizophrenic patients relative to chronic schizophrenic patients and healthy controls. This suggests that reduced brain antioxidant enzyme levels may contribute to oxidative damage in acute schizophrenia [210], though larger studies are needed to confirm this finding.

Several additional experimental and human studies examined in more detail the mechanisms underlying the pathophysiology of increased oxidative stress in psychiatric disorders [206-262]. In animal models of depression, brain levels of glutathione are reduced while lipid peroxidation and NO levels are increased [206,262].

Postmortem studies show reduced brain levels of total glutathione in MDD, BPD [206] and schizophrenic subjects [206,207]. Fibroblasts cultured from MDD patients show increased oxidative stress independent of glutathione levels [262], arguing against a primary role of glutathione depletion as the major mechanism of oxidative stress in depression.

Microglial activation may increase oxidative stress through its production of proinflammatory cytokines and NO [206-209]. Proinflammatory cytokines and high NO levels may promote reactive oxygen species (ROS) formation, which in turn accelerates lipid peroxidation, damaging membrane phospholipids and their membrane-bound monoamine neurotransmitter receptors and depleting endogenous antioxidants. Increased ROS products can enhance microglial activation and increase proinflammatory production via stimulating NF-κB [208], which in turn perpetuates oxidative injury [208], creating the potential for a pathological positive feedback loop in some psychiatric disorders [206-209]. Although neuroinflammation can increase brain glutamate levels [85,86], the role of glutamatergic hyperactivity as a cause of oxidative stress remains unsubstantiated [207].

Mitochondrial dysfunction may contribute to increased oxidative stress in MDD, BPD and schizophrenia [206]. Postmortem studies in these disorders reveal abnormalities in mitochondrial DNA, consistent with the high prevalence of psychiatric disturbances in primary mitochondrial disorders [206]. *In vitro* animal studies show that proinflammatory cytokines, such as TNF-α, can reduce mitochondrial density and impair mitochondrial oxidative metabolism [211,212], leading to increased ROS production [206,213]. These experimental findings may imply mechanistic links among neuroinflammation, mitochondrial dysfunction and oxidative stress [206,213], meriting further investigation of these intersecting pathogenic pathways in human psychiatric disorders.

The vulnerability of neural tissue to oxidative damage varies among different psychiatric disorders based on the neuroanatomical, neurochemical and molecular pathways involved in the specific disorder [207]. Treatment effects may also be critical, as preliminary evidence suggests that antipsychotics, SSRIs and mood stabilizers possess antioxidant properties [206,207,262]. The therapeutic role of adjuvant antioxidants (for example, vitamins C and E) in psychiatric disorder remains to be substantiated by high-powered randomized clinical trials. N-acetylcysteine shows the most promising results to-date, with several randomized placebo-controlled trials demonstrating its efficacy in MDD, BPD and schizophrenia [207].

#### Blood–brain barrier dysfunction

The BBB secures the brain’s immune-privileged status by restricting the entry of peripheral inflammatory mediators, including cytokines and antibodies that can impair neurotransmission [214,215]. The hypothesis of BBB breakdown and its role in some psychiatric patients [60,214,216,217] is consistent with the increased prevalence of psychiatric comorbidity in diseases associated with its dysfunction, including SLE [97], stroke [11], epilepsy [218] and autoimmune encephalitides (Table 1). An elevated ‘CSF:serum albumin ratio’ in patients with MDD and schizophrenia suggests increased BBB permeability [214].

In one study (63 psychiatric subjects, 4,100 controls), CSF abnormalities indicative of BBB-damage were de-tected in 41% of psychiatric subjects (14 MDD and BPD, 14 schizophrenia), including intrathecal synthesis of IgG, IgM, and/or IgA, mild CSF pleocytosis (5 to 8 cells per mm^3^) and the presence of up to four IgG oligoclonal bands [216]. One post-mortem ultrastructural study in schizophrenia revealed BBB ultrasructural abnormalities in the prefrontal and visual cortices, which included vacuolar degeneration of endothelial cells, astroglial-end-foot-processes, and thickening and irregularity of the basal lamina [60]. However, in this study, the authors did not comment on the potential contribution of postmortem changes to their findings. Another study investigating transcriptomics of BBB endothelial cells in schizophrenic brains identified significant differences among genes influencing immunological function, which were not detected in controls [217].

Oxidation-mediated endothelial dysfunction may contribute to the pathophysiology of BBB dysfunction in psychiatric disorders. Indirect evidence from clinical and experimental studies in depression [219] and, to a lesser extent, in schizophrenia [220] suggests that increased oxidation may contribute to endothelial dysfunction. Endothelial dysfunction may represent a shared mechanism accounting for the known association between depression and cardiovascular disease [219,221], which may be related to decreased levels of vasodilator NO [221-223]. Experimental studies suggest that reduced endothelial NO levels are mechanistically linked to the uncoupling of endothelial nitric oxide synthase (eNOS) from its essential co-factor tetrahydrobiopterin (BH4), shifting its substrate from L-arginine to oxygen [224-226]. Uncoupled eNOS promotes synthesis of ROS (for example, superoxide) and reactive nitrogen species (RNS) (for example, peroxynitrite; a product of the interaction of superoxide with NO) [227] rather than NO, leading to oxidation-mediated endothelial dysfunction [224-226].

Animal data showed that SSRIs could restore deficient endothelial NO levels [219], suggesting that anti-oxidative mechanisms may contribute to their antidepressant effects. In humans, L-methylfolate may potentiate antidepressant effects of SSRIs [228], putatively by increasing levels of BH4, which is an essential cofactor for eNOS re-coupling-mediated anti-oxidation [229], as well as for the rate-limiting enzymes of monoamine (that is, serotonin, norepinephrine, dopamine) synthesis [228].

Taken together, both the recent work emphasizing the role of uncoupled eNOS-induced oxidative stress in the pathogenesis of vascular diseases [230,231] and the epidemiological studies establishing depression as an independent risk factor for vascular pathologies, such as stroke and heart disease [219,221], add further support to the clinical relevance of uncoupled eNOS-mediated endothelial oxidative damage in depression. Despite abundant evidence for cytokine abnormalities in human psychiatric illnesses and the experimental data showing that proinflammatory cytokines can reduce eNOS expression [212] and increase BBB permeability [215], human evidence that directly links excess proinflammatory cytokines to eNOS dysfunction and/or BBB impairment is lacking.

### Imaging and treating inflammation in psychiatric illness

#### Imaging neuroinflammation in situ

Clinically, neuroinflammation imaging may prove to be crucial for identifying the subgroup of psychiatric patients with neuroinflammation who would be most likely to respond favorably to immunomodulatory therapies. Additionally, such imaging may allow clinicians to monitor neuroinflammation-related disease activity and its response to immune therapy in psychiatric patients. Imaging inflammation in the human brain has traditionally depended upon MRI or CT visualization of extravagated intravenous contrast agents, indicating localized breakdown of the BBB. Gadolinium-enhanced MRI occasionally demonstrates such breakdown in the limbic regions associated with emotional processing in patients with psychiatric disorders attributable to paraneoplastic or other encephalitides [107,109,113]. To our knowledge, however, abnormal enhancement has never been demonstrated in any classical psychiatric disorder [21,214,232], despite functional [214,216] and ultrastructural BBB abnormalities [60].

Whether or not subtler neuroinflammation can be visualized *in vivo* in classical psychiatric disorders remains unknown. One promising technique is positron emission tomography (PET) using radiotracers, such as C11-PK11195, which bind to the translocator protein, previously known as the peripheral benzodiazepine receptor, expressed by activated microglia [233,234].

Using this method, patients with schizophrenia were shown to have greater microglial activation throughout the cortex [235] and in the hippocampus during acute psychosis [236]. One study (14 schizophrenia, 14 controls) found no significant difference between [11C] DAA1106 binding in schizophrenia versus controls, but a direct correlation between [11C] DAA1106 binding and the severity of positive symptoms and illness duration in schizophrenia [236].

Investigators from our institution utilized C11-PK11195 PET to demonstrate bi-hippocampal inflammation in a patient with neuropsychiatric dysfunction, including psychotic MDD, epilepsy, and anterograde amnesia, associated with anti-GAD antibodies [237]. However, PK11195 PET has low signal-to-noise properties and requires an on-site cyclotron.

Accordingly, research is being devoted to developing improved translocator protein ligands for PET and SPECT. Future high-powered post-mortem brain tissues studies utilizing protein quantification aimed at elucidating metabolic and inflammatory pathways, CNS cytokines and their binding receptors, in psychiatric disorders are needed to advance our understanding of the autoimmune pathophysiology.

#### Role of antiinflammatory drugs in psychiatric disorders

Several human and animal studies suggest that certain antiinflammatory drugs may play an important adjunctive role in the treatment of psychiatric disorders (Table 3). Common drugs are cyclooxygenase inhibitors (Table 3) [238-245], minocycline (Table 3) [240-245], omega-3 fatty acids [246,247], and neurosteroids [248].

**Table 3:** Selected studies assessing the efficacy of antiinflammatory agents among patients with unipolar and bipolar depression, schizophrenia, and obsessive-compulsive disorder

**Study**	**Study design**	**Group comparison**	**Number of subjects**	**Functional outcome**
**COX-2 inhibitors**				
Müller et al. 2006 [238]	RCT	Celecoxib (200 mg bid) + reboxetine versus placebo + reboxetine	40 MDD (acute)	Significantly greater decrease in depressive symptoms in the treatment group (P = 0.035)
Akhondzadeh et al. 2009 [239]	RCT	Celecoxib (200 mg bid) + fluoxetine versus placebo + fluoxetine	40 MDD	Significant improvement of depressive symptoms (P <0.001), and a greater percentage of responders (90% versus 50%, P = 0.01) and remission (35% versus 5%, P = 0.04) in the treatment group
Medlewicz et al. 2006 [240]	Open-label	Acetylsalicylic acid (160 mg qd) + SRI	24 MDD and BPD	52.4% responder rate, significant improvement within one week (P <0.0001) following treatment; sustained at four weeks
ClinicalTrials.gov http://www.clinicaltrials.gov/NCT00510822 completed, pending results	RCT	Cimicoxib (50 mg bid) + sertraline versus placebo + sertraline	169 MDD	Primary outcome measure is mean change in Hamilton Depression Rating Scale from baselineto six-week endpoint
Nery et al. 2008 [263]	RCT	Celecoxib versus placebo	28 BPD (depressive and mixed states)	No significant differences in depressive or manic symptoms.
Müller et al. 2002 [242]	RCT	Celecoxib (400 mg qd) + risperidone versus placebo + risperidone	50 (acute schizophrenia)	Significant improvement of positive and negative symptoms (P = 0.05), as well as cognition (P <0.06) in treatment group at five weeks
Müller et al. 2010 [244]	RCT	Celecoxib + amisulpride versus placebo + amisulpride	49 schizophrenia (first-episode)	Significant improvement of positive and negative symptoms in celecoxib plus amisulpride group relative to amisulpride alone (P <0.001) at six-weeks
Sayyah et al. 2011 [245]	RCT	Celecoxib (200 mg bid) + fluoxetine versus placebo + fluoxetine	50 OCD	Significantly greater reduction in YBOCS scores in the celecoxib treatment group at two weeks (P = 0.007) and at the eight week end-point (P = 0.037)
**Minocycline**				
Levine et al. 1996 [264]	Case report	Minocycline (150 mg qd) started 20 years after disease onset	1 BPD	Marked decrease in depressive symptoms (HAM-D score went from 25 to 8) within one week following treatment, sustained at two weeks.
Levkovitz et al. 2009 [265]	RCT	Minocycline (200 mg qd) versus placebo	21 schizophrenia (early and acute-phase)	Significant improvement of negative symptoms and cognitive dysfunction in treatment group (P <0.01)
ClinicalTrials.gov NCT01433055 recruiting, estimated completion 7/15	RCT	Minocycline (100 mg bid) + clozapine versus placebo + clozapine	60 schizophrenia (refractory to ≥2 antipsychotics)	Primary outcome is the improvement in positive symptoms as measured by the four-item sub-factor of the Brief Psychiatric Rating Scale.
Miyaoka et al. 2007 [266]	Case series	Minocycline (150 mg qd) + stable antipsychotic regiment	2 schizophrenia	Complete resolution of positive and negative symptoms with minocycline, sustained for one to two years. Symptom exacerbation occurred one-week following minocycline discontinuation (in both cases). In one patient, the complete resolution of symptoms occurred at age 61, which was 41 years after disease onset.
Miyaoka et al. 2008 [267]	Open-label	Minocycline (150 mg tid)	22 schizophrenia	Significant improvement of positive and negative symptoms at four to eight weeks (P = 0.0001)
Rodriguez et al. 2010 [268]	Open-label	Minocycline (100 mg bid)	9 OCD	22% had a 40% to 46% YBOCS reduction at 12 weeks; the group as a whole did not have a significant change in YBOCS score.

*ASA*, acetylsalicylic acid; *BPD*, bipolar disorder; *COX-2*, cycloxygenase-2; *HAM-D*: Hamilton Depression Rating Scale; *MDD*, major depressive disorder; *OCD*, obsessive-compulsive disorder; *SRI*, serotonin reuptake inhibitor; *RCT*, randomized controlled trial; *YBOCS*: Yale-Brown Obsessive-Compulsive Scale.

Several human studies showed that COX-2 inhibitors could ameliorate psychiatric symptoms of MDD, BPD, schizophrenia and OCD (Table 3) [248]. By contrast, adjunctive treatment with non-selective COX-inhibitors (that is, non-steroidal antiinflammatory drugs (NSAIDs)) may reduce the efficacy of SSRIs [249,250]; two large trials reported that exposure to NSAIDs (but not to either selective COX-2 inhibitors or salicylates) was associated with a significant worsening of depression among a subset of study participants [249,250].

In the first trial, involving 1,258 depressed patients treated with citalopram for 12 weeks, the rate of remission was significantly lower among those who had taken NSAIDs at least once relative to those who had not (45% versus 55%, OR 0.64, *P* = 0.0002) [249]. The other trial, involving 1,545 MDD subjects, showed the rate of treatment-resistant depression was significantly higher among those taking NSAIDs (OR 1.55, 95% CI 1.21 to 2.00) [231]. The worsening of depression in the NSAID groups may not be mechanistically linked to NSAID therapy but instead related to co-existing chronic medical conditions [10,12-18] that necessitate long-term NSAIDs and which are known to be independently associated with increased risk of treatment-resistant depression [249,251]. Future studies investigating the impact of NSAIDs on depression and response to antidepressants in humans are needed.

In other experimental studies utilizing acute-stress paradigms to induce a depression-like state in mice, citalopram increased TNF-α, IFN-γ, and p11 (molecular factor linked to depressive behavior in animals) in the frontal cortex, while the NSAID ibuprofen decreased these molecules; NSAIDs also attenuated the antidepressant effects of SSRIs but not other antidepressants [249]. These findings suggest that proinflammatory cytokines may paradoxically exert antidepressant effects despite overwhelming evidence from human studies to the contrary (as reviewed above), which can be attenuated by NSAIDs [249]. At least two considerations may account for this apparent paradox: 1) under some experimental conditions, proinflammatory cytokines have been associated with a neuroprotective role, [251; (for example, IFN-γ in low levels can induce neuroprotective microglia (Figure 2) [163,166,251]); and 2) whether these responses observed in the context of an acute stress paradigm in an animal model are applicable to endogenous MDD in humans remains unclear [251].

The therapeutic effects of COX-2 inhibitors in psychiatric disorders may involve modulation of biosynthesis of COX-2-derived prostaglandins, including proinflammatory PGE2 and antiinflammatory 15-deoxy-Δ^12,14^-PGJ_2_ (15d-PGJ_2_) [252,253]. COX-2 inhibitors can reduce PGE2-mediated inflammation, which may contribute to the pathophysiology of psychiatric disorders [252,253]. They may also alter the levels 15d-PGJ_2_, and the activity of its nuclear receptor peroxisome proliferator-activated nuclear receptor gamma (PPAR-γ) [252,253].

Several studies suggest that 15d-PGJ_2_ and its nuclear receptor PPAR-γ can serve as biological markers for schizophrenia [253]. In schizophrenic patients, serum PGE2 levels are increased, whereas serum levels of 15d-PGJ_2_ are decreased, as is the expression of its nuclear receptor PPAR-γ in PBMC [252]. While COX-2 inhibitors may limit the potentially beneficial antiinflammatory effects of the COX-2–dependent ‘15d-PGJ_2_/PPAR-γ pathway’, they may advantageously reduce its harmful effects, including 1) the increased risk for myocardial infarction and certain infections (for example, cytomegalovirus and *Toxoplasma gondii*) in schizophrenic patients [254] and 2) its pro-apoptotic effects observed in human and animal cancer tissue [255]. Other potential mechanisms of COX-2 inhibitors therapeutic effects may involve their ability to reduce proinflammatory cytokine levels [163], limit quinolinic acid excitotoxicity (as in MDD) and decrease KYNA levels (as in schizophrenia) [128].

Minocycline can be effective in psychiatric disorders (Table 3) [248]. *In vitro* data suggest that minocycline inhibits MAP, cytokine secretion, ‘COX-2/PGE-2 expression,’ and inducible nitric oxide synthase [256]. Minocycline may also counteract dysregulated glutamatergic and dopaminergic neurotransmission [256].

Omega-3 fatty acid effectiveness in psychiatric disorders is unclear [248]. In a 2011 meta-analysis of 15 randomized-controlled trials (916 MDD), omega-3 supplements containing eicosapentaenoic acid ≥60% (dose range 200 to 2,200 mg/d in excess of the docosahexaenoic acid dose) significantly decreased depressive symptoms as an adjunctive therapy to SRIs (*P* <0.001) [246]. A subsequent meta-analysis, however, concluded that there is no significant benefit of omega-3 fatty acids in depression and that the purported efficacy is merely a result of publication bias [247]. A 2012 meta-analysis of 5 randomized-controlled trials including 291 BPD participants found that depressive, but not manic, symptoms were significantly improved among those randomized to omega-3 fatty acids relative to those taking placebo (Hedges g 0.34, *P* = 0.025) [257]. In a randomized controlled trial of schizophrenic subjects followed up to 12 months, both positive and negative symptom scores were significantly decreased among the 66 participants randomized to long-chain omega-3 (1.2 g/day for 12 weeks; *P* = 0.02 and 0.01, respectively) [258]; the authors concluded that omega-3 augmentation during the early course of schizophrenia can also prevent relapses and disease progression [258].

A 2012 meta-analysis of seven randomized-controlled trials assessing omega-3 augmentation in 168 schizophrenic patients found no benefit of treatment [259]. The authors of this meta-analysis specifically stated that no conclusion could be drawn regarding the relapse prevention or disease progression endpoints [259]. Experimental data suggest that eicosapentaenoic acid and docosahexaenoic acid mediate their antiinflammatory effects by promoting synthesis of resolvins and protectins, which can inhibit leukocyte infiltration and reduce cytokine production [248].

Neurosteroids, including pregnenolone and its downstream metabolite allopregnanolone, may have a beneficial role in some psychiatric disorders [248,260]. In MDD, several studies found decreased plasma/CSF allopregnanolone levels correlating with symptom severity, which normalized after successful treatment with certain antidepressants (for example, SSRIs), and electroconvulsive therapy [261]. In schizophrenia, brain pregnenolone levels can be altered [248] and serum allopregnanolone levels may increase after some antipsychotic drugs (for example, clozapine and olanzapine) [260]. In three randomized-controlled trials (100 schizophrenia (pooled); treatment duration, approximately nine weeks) positive, negative, and cognitive symptoms, as well as extrapyramidal side effects of antipsychotics were significantly improved in one or more trials among those randomized to pregnenolone relative to those receiving placebo [248]. In one trial, the improvement was sustained with long-term pregnenolone treatment [248]. Pregnenolone can regulate cognition and behavior by potentiating the function of NMDA and GABA_A_ receptors [248]. Furthermore, allopregnanolone may exert neuroprotective and antiinflammatory effects [248]. More RCT studies are needed to confirm the beneficial role of neuroactive steroids in early-onset psychiatric disorders in humans.

We are awaiting the results of several ongoing clinical trials investigating the therapeutic effects of other anti-inflammatory agents, including salicylate, an inhibitor of NF-κB (NCT01182727); acetylsalicylic acid (NCT01320982); pravastatin (NCT1082588); and dextromethorphan, a non-competitive NMDAR antagonist that can limit inflammation-induced dopaminergic neuronal injury (NCT01189006).

#### Future treatment strategies

Although current immune therapies (for example, IVIG, plasmapheresis, corticosteroids and immunosuppressive agents) are often effective for treating autoimmune encephalitides wherein inflammation is acute, intense and predominately of adaptive origin, their efficacy in classical psychiatric disorders wherein inflammation is chronic, much milder, and predominately of innate origin, is limited [2]. Development of novel therapeutics should aim at reversing glial loss [46,138], down-regulating harmful MAP, while optimizing endogenous neuroprotective T regs and beneficial MAP, rather than indiscriminately suppressing inflammation as occurs with current immunosuppressive agents. Additionally, development of potent co-adjuvant antioxidants that would reverse oxidative injury in psychiatric disorders is needed.

## Conclusions

Autoimmunity can cause a host of neuropsychiatric disorders that may initially present with isolated psychiatric symptoms. Innate inflammation/autoimmunity may be relevant to the pathogenesis of psychiatric symptoms in a subset of patients with classical psychiatric disorders. Innate inflammation may be mechanistically linked to the traditional monoaminergic and glutamatergic abnormalities and increased oxidative injury reported in psychiatric illnesses.

## Abbreviations

3-OH-KYN: 3-hydroxy-kynurenine; α7nAchR: Alpha 7 nicotinic acetylcholine receptors; AMPAR: Amino-3-hydroxy-5-methyl-l-4-isoxazolepropionic acid receptors; APC: Antigen presenting cell; BBB: Blood–brain barrier; BH4: Tetrahydrobiopterin; BPD: Bipolar disorder; CI: Confidence interval; CNS: Central nervous system; COX-2: Cyclooxegenase-2; CSF: Cerebrospinal fluid; DSM-IV: Diagnostic and Statistical Manual of Mental Disorders 4th Edition; EAATs: Excitatory amino acid transporters; eNOS: Endothelial nitric oxide synthase; GABAB: Gamma aminobutyric acid-beta; GAD: Glutamic acid decarboxylase; GFAP: Glial fibrillary acidic protein; GLX: ^1^H MRS detectable glutamate, glutamine, gamma aminobutyric acid composite; IDO: Indoleamine 2,3-dioxygenase; Ig: Immunoglobulin; IL: Interleukin; IL-1RA: Interleukin 1 receptor antagonist; IFN-γ: Interferon gamma; KAT: Kynurenine aminotransferase; KMO: Kynurenine 3-monooxygenase; KYN: Kynurenine; KYNA: Kynurenic acid; LE: Limbic encephalitis; LPS: Lipopolysaccharide; MAP: Microglial activation and proliferation; MDD: Major depressive disorder; mGluR: Metabotropic glutamate receptor; MHC: II Major histocompatibility complex class two; MRI: Magnetic resonance imaging; MRS: Magnetic resonance spectroscopy; NF-κB: Nuclear factor kappa B; NMDAR: N-methyl-D-aspartate receptor; NR1: Glycine site; OCD: Obsessive-compulsive disorder; OR: Odds ratio; PANDAS: Pediatric neuropsychiatric autoimmune disorders associated with streptococcal infections; PBMC: Peripheral blood mononuclear cells; PET: Positron emission tomography; PFC: Prefrontal cortex; PGE-2: Prostaglandin E2; PPAR-γ: Peroxisome proliferator-activated nuclear receptor gamma; QA: Quinolinic acid; RNS: Reactive nitrogen species; ROS: Reactive oxygen species; sIL: Soluble interleukin; SLE: Systemic lupus erythematosus; SRI: Serotonin reuptake inhibitor; TNF-α: Tumor necrosis factor alpha; T-regs: CD4^+^CD25^+^FOXP3^+^ T regulatory cells; TDO: Tryptophan-2,3-dioxygenase; Th: T-helper; VGKC: Voltage-gated potassium channel; XAG-: Glutamate aspartate transporter; Xc-: Sodium-independent astroglial glutamate/cystine antiporter system

## Competing interests

The authors declare that they have no competing interests.

## Authors’ contributions

SN and DMP performed an extensive literature review, interpreted data, prepared the manuscript, figures, and tables. KA prepared the section pertaining to oxidative mechanisms and contributed to the manuscript revisions. AN and OD critically-revised and improved the design and quality of the manuscript. All authors read and approved the final manuscript.
